# Anthocyanin-Incorporated Chromogenic Agar for Rapid, Selective Detection of *Streptococcus pneumoniae* via Hydrogen Peroxide-Mediated Oxidation

**DOI:** 10.1021/acsomega.5c13596

**Published:** 2026-03-02

**Authors:** Cagla Celik Yoldas, Nimet Temur, Nilay Ildiz, Pinar Sagiroglu, Mustafa Altay Atalay, Ismail Ocsoy

**Affiliations:** † Department of Analytical Chemistry, Faculty of Pharmacy, 52966 Harran University, 63200 Sanliurfa, Türkiye; ‡ Department of Analytical Chemistry, Faculty of Pharmacy, 52958 Erciyes University, 38039 Kayseri, Türkiye; § Medical Imaging Department, Vocational School of Health Services, 450200 Bandirma Onyedi Eylul University, 10200 Balikesir, Türkiye; ∥ Department of Medical Microbiology, Faculty of Medicine, 52958 Erciyes University, 38039 Kayseri, Türkiye

## Abstract

The rapid and cost-effective identification of *Streptococcus pneumoniae* (*S. pneumoniae*) is crucial for the prompt treatment of pneumonia and meningitis. However, conventional culture methods are time-consuming and molecular techniques are prohibitively expensive in resource-limited settings. To address this challenge, we developed a novel anthocyanin chromogenic agar as a diagnostic tool for the selective and rapid detection of *S. pneumoniae*. Unlike traditional pH-based indicators, this method exploits the unique oxidative capability of *S. pneumoniae*, which releases excessive amounts of hydrogen peroxide (H_2_O_2_). This results in the oxidative degradation of the blue anthocyanin pigment to a colorless form, creating distinct gray zones. The detection efficiency was evaluated as a function of bacterial concentration and incubation time. Upon inoculation at concentrations ranging from 1 to 1000 CFU/mL, color changes were monitored visually over 24 h. Notably, an inoculum of 1000 CFU/mL produced visible gray spots within just 7 h of incubation. The anthocyanin chromogenic agar demonstrated high selectivity against common coexisting pathogens, which caused a pH-dependent color change from blue to pink, whereas only *S. pneumoniae* induced the specific gray signal. Furthermore, colorimetric results were analyzed using digital image processing for objective detection and differentiation. In conclusion, anthocyanin chromogenic agar presents a clinically applicable alternative that significantly reduces both incubation time and cost compared to other chromogenic agars. While the standardization of natural anthocyanin stability remains a consideration for commercial upscaling, this method offers a promising perspective for decentralized testing in resource-limited settings.

## Graphical abstract

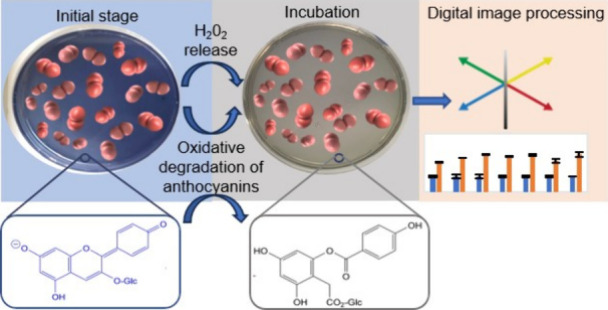

## Research Highlights

- A novel anthocyanin chromogenic agar was developed for the selective identification of *Streptococcus pneumoniae*.
- The detection mechanism is based on the specific oxidative degradation of anthocyanins by bacterial hydrogen peroxide.
- *Streptococcus pneumoniae* colonies produce distinct colorless/gray zones, distinguishing them from acid-producing pathogens (pink).
- The method enables rapid visual detection within 7 h, significantly reducing the diagnosis time compared to standard culture.
- This natural pigment-based medium offers a cost-effective and accessible alternative to synthetic commercial agars.

## 1 Introduction

*Streptococcus pneumoniae* (*S. pneumoniae*) is a common bacterium that causes infection in the human lower respiratory system and causes both invasive (e.g., pneumonia, meningitis, bacteremia) and noninvasive (e.g., acute otitis media, sinusitis) diseases. According to epidemiological studies, approximately 10% of adults and between 27% and 65% of children are carriers of *S. pneumoniae*.^1,2^ World Health Organization (WHO) has classified *S. pneumoniae* as one of 12 priority pathogens since 2017.^3^ The WHO has published the 2024 Bacterial Priority Pathogens List (BPPL) including macrolide-resistant Group A Streptococci and penicillin-resistant Group B Streptococci for the first time. In addition, the WHO BPPL identified *S. pneumoniae* as one of the 24 priority pathogens requiring urgent new antibiotics.^4^ *S. pneumoniae* infections primarily affect young children, immunosuppressed patients and the geriatric population. *S. pneumoniae* causes approximately 15 million illnesses and leads to over one million pneumonia-related deaths worldwide each year. Pneumonia is an infectious disease that affects the lung tissue that causes hemorrhaging, inflammation and damage to the lung tissue. If treatment is delayed or inadequate, the infection can enter the bloodstream, leading to septic shock, multiple organ failure, cardiotoxicity and death within a few days of the initial symptoms appearing.^5–8^ *S. pneumoniae* produces high levels of endogenous hydrogen peroxide (H_2_O_2_) due to its catalase negativity, as a result of lactate oxidase and pyruvate oxidase enzymatic activity. The high levels of H_2_O_2_ released by *S. pneumoniae* into the alveolar sacs can cause fatal hypoxaemia by impairing gas exchange due to disruption of the Na^+^–K^+^ pump. Furthermore, endogenous H_2_O_2_ can rapidly diffuse across cell membranes and accumulate in the extracellular environment of pneumococci to inhibit other pathogens.^1,9,10^ Furthermore, *S. pneumoniae* induces significant DNA damage and apoptosis in alveolar epithelial cells through two key virulence factors, H_2_O_2_ and pneumolysin.^11–13^ Although it is known in the literature that different bacterial species can produce H_2_O_2_, it has been shown that *S. pneumoniae* is distinguished from many other pathogens by its exceptionally high H_2_O_2_ production at levels of 1–3 mM under aerobic conditions.^14–16^ These values are quite high compared to many bacterial species, and considering that the H_2_O_2_ produced by pneumococci can cause genotoxic/oxidative damage to host cells and is at a level that can be used for diagnostic purposes, it provides a suitable biochemical basis for developing colorimetric sensors.

Early, reliable, and accurate detection of *S. pneumoniae* is crucial in the combating of infection. For this purpose, morphological and biochemical tests (both routine and advanced) are used to diagnose *S. pneumoniae*. Gram staining and sputum culture are the initial diagnostic steps for identifying *S. pneumoniae*. Traditional culture-based diagnostic methods for cerebrospinal fluid, blood or respiratory tract samples remain the gold standard for diagnosis. Currently, the gold standard for the identification of *S. pneumoniae* involves culture on blood agar followed by Optochin susceptibility and bile solubility tests. However, these conventional methods are labor-intensive and typically require 24–48 hours (h) to yield results.^17,18^ There are also several ways to detect *S. pneumoniae* that are not cultural methods, such as mass spectrometry, polimerase chain reaction, and immunoassay. While these tests offer significant time savings compared to the gold-standard culture-based methods, they are not suitable for widespread use due to the need for technical infrastructure, specialized personnel, and expensive equipment and devices.^19–21^ Access to reliable microbiological diagnostic tests is limited in middle- and low-income countries. This can lead to inaccurate or delayed diagnoses, inadequate treatment, increased mortality rates and an increased prevalence of disease.^22^ Therefore, rapid, sustainable and economical phenotypic culture-based diagnostic methods remain an attractive alternative.

Chromogenic agar medias are an accurate, rapid and economical alternative that improves the effectiveness of culture-based tests. In recent years, the use of chromogenic agar media has grown substantially in clinical microbiology. These agars typically combine selective agents with chromogenic enzyme substrates, which are then metabolized (often hydrolyzed) by target organisms to form colonies with characteristic colors. This enables simultaneous selectivity and direct visual differentiation in the primary culture. As Perry’s review shows, the range of commercially available chromogenic agar media has grown markedly since the mid-2000s, extending beyond traditional applications to include additional clinically relevant pathogens such as *Pseudomonas aeruginosa* (*P. aeruginosa*), group B streptococci, *Clostridioides difficile*, *Campylobacte*r spp. and *Yersinia enterocolitica*, as well as media designed for screening acquired antimicrobial resistance mechanisms such as vancomycin-resistant *Enterococci* (VRE) and carbapenemase/extended-spectrum β-lactamase (ESBL) producers. Overall, color-based colony recognition can reduce the number of colonies requiring further analysis in polymicrobial cultures and streamline culture-based diagnostics. Chromogenic agar media represent a significant innovation in culture-based diagnostic methods. They eliminate the need for additional biochemical testing, enabling clinicians to make an early diagnosis and initiate antibiotic treatment.^23,24^ Recent studies have demonstrated the operational advantages of chromogenic media over traditional methods. For instance, Khutade et al. compared the performance of HiCrome UTI agar with conventional media (MacConkey, Blood, and CLED agar) for urinary tract isolates, reporting that the chromogenic medium provided the highest sensitivity (100% growth) while significantly reducing the laboratory workload.^25^ Furthermore, selective chromogenic media have been successfully developed for the rapid detection of specific resistance phenotypes, particularly in *P. aeruginosa*. Rosa et al. evaluated the BIChromET selective medium for detecting piperacillin-tazobactam and cefepime-resistant *P. aeruginosa* in respiratory specimens, documenting high accuracy (92.6%–100% agreement) compared to reference liquid microdilution methods.^26^ Similarly, Mairal et al. reported that their novel selective medium for meropenem-resistant *P. aeruginosa* achieved 98.7% sensitivity and 92.3% categorical agreement within 24 h.^27^

However, despite these successes with common pathogens, significant limitations remain regarding fastidious organisms. Rizwana et al. noted that while HiCrome agar identified key pathogens such as *Escherichia coli* (*E. coli*), *Klebsiella pneumoniae* (*K. pneumoniae*), and *Enterococcus faecium* (*E. faecium*) with 100% accuracy, it showed limited success in detecting fastidious organisms from pyogenic infections.^28^ More critically in the context of respiratory pathogens, Robberts et al. evaluated the antimicrobial susceptibility and identification performance of Chromatic MH agar for direct disk diffusion from positive blood cultures. They were able to identify *Staphylococcus aureus*, *Streptococcus pyogenes*, *Streptococcus agalactiae*, *Listeria monocytogenes*, *E. coli*, *Shigella sonnei*, *Citrobacter freundii*, *K.* *pneumoniae* and *P.* aeruginosa, *Salmonella*, *Acinetobacter*, *Burkholderia*, and *Yersinia enterocolitica*. They explicitly reported that the medium was inadequate for recovering *S. pneumoniae* and that it required further optimization.^29^ This highlights a critical gap in current chromogenic technology for the rapid and reliable detection of pneumococcal infections.

Our research group has previously presented a chromogenic, agar-like medium and liquid tests containing anthocyanin molecule as a pH indicator.^30–33^ The main component of this medium is anthocyanin, which change color in the presence of acidic components released as a result of bacterial metabolic activity. These molecules act as natural pH indicators and exhibit a broad color spectrum ranging from pink to purple/blue and yellow in acidic, neutral or alkaline environments. Anthocyanins are natural pH indicators that can become colorless or gray when degraded by light, heat, copigmentation, sulphites, ascorbic acid, oxygen and enzymes. A colorless appearance is particularly evident because of strong oxidative degradation with H_2_O_2_.^33–36^ In summary, the current diagnostic methods have significant limitations. Traditional culture methods are inherently time-consuming and labor-intensive, while molecular techniques require sophisticated infrastructure, specialized personnel and expensive equipment. Furthermore, while commercial chromogenic agars offer a simplified detection workflow, they predominantly rely on synthetic enzyme substrates, which increase production costs significantly.^17–24^ Therefore, there is an urgent need for the development of rapid, selective, and cost-effective diagnostic tools for *S. pneumoniae* detection that utilize natural indicators, such as anthocyanins, to provide an affordable alternative for routine clinical screening.

Herein, we demonstrate that the high secretion of H_2_O_2_ by *S. pneumoniae* induces the oxidative degradation of anthocyanin molecules in the agar plate. The color of the *S. pneumoniae* inoculated agar plate changes from purple to gray as a result of H_2_O_2_-mediated anthocyanin oxidation. This mechanism is distinct from our previous findings, where the agar shifted from purple to pink due to the release of acidic volatile metabolites. This is the first report demonstrating that H_2_O_2_ is a key virulence factor of *S. pneumoniae* drives this specific colorimetric change in agar plate. The oxidation of anthocyanin results in its conversion to a gray and even colorless forms. However, the gray appearance observed on chromogenic agar is due to components of the agar, such as beef extract and peptone. We quantified these agar-based results using digital image processing, specifically Red Green Blue (RGB) and total color difference (Delta E/ΔE) analyses. This chromogenic agar is considered an important innovation for culture-based diagnostic methods as it is thought to reduce the time required for traditional methods. The development of anthocyanin chromogenic agar has paved the way for a new era in clinical laboratories. This innovative solution allows for the simultaneous execution of both culture and diagnosis processes, eliminating the need for additional biochemical diagnostic tests. We anticipate that this proposed chromogenic agar will serve as a rapid, cost-effective, and direct tool for pathogen detection in clinical settings.

## 2 Results and Discussion

Our team had previously prepared red cabbage extract (RCE) agar for the first time using anthocyanin-rich RCE as a natural pH indicator and an alternative to chromogenic agents. RCE contains anthocyanins giving different colors in acidic, neutral and alkaline environments based upon their protonated or deprotonated forms.^37,38^ Our team has demonstrated that meticillin resistant bacterias, release organic volatile compounds during cultivation.^39,40^ The action mechanism of RCE agar was based on protonation of anthocyanins by acidic volatile organic compounds released methicillin-resistant*Staphylococcus aureus* (MRSA) and methicillin-resistant *Staphylococcus epidermidis* (MRSE) cultured on the agar. Although the original color was purple-blue, the protonated anthocyanins gave pink, which could indicate the growth of methicillin-resistant pathogens.^32^

In this study, we developed a new, rapid, sensitive, and accurate chromogenic anthocyanin agar for selective detection of *S. pneumoniae*. The main component of the recommended chromogenic anthocyanin agar is the anthocyanin molecule. We investigated the H_2_O_2_-mediated oxidative degradation of anthocyanins rather than their pH indicator properties. Compared to other bacterial agents, *S. pneumoniae* produces excessive amounts of H_2_O_2_, which is a known virulence factor. H_2_O_2_ triggers the oxidative degradation of anthocyanins, converting them to a colorless form. Here, we demonstrate that H_2_O_2_, released by *S. pneumoniae*, diffuses through chromogenic anthocyanin agar and strongly oxidizes anthocyanins. After incubation, the blue colored agar plate turns gray due to the conversion of the anthocyanin to its colorless form.

The potential color change mechanism of the chromogenic agar is based on the degradation of the anthocyanin molecule in the presence of H_2_O_2_, as shown in the Scheme 1. A possible oxidation mechanism of anthocyanin by H_2_O_2_ has been proposed in various studies. According to this mechanism, anthocyanins treated with H_2_O_2_ undergo oxidative degradation to form a highly unstable, colorless hemiketal structure. Rearrangement of this hemiketal structure leads to the formation of a glucose ester. Thus, the observed gray spots represent the zones where complete anthocyanin degradation has occurred, revealing the colorless background of the agar matrix.^41–43^ Figure S1 shows the results of the interaction between H_2_O_2_ at different concentrations (0.1–1000 mM) and anthocyanin solutions. Complete oxidative degradation occurred immediately at 1000 mM, within 5 min at 100 mM, and after 30 min at 10 mM. Additionally, a gray color appeared in the solution containing 1 mM H_2_O_2_ after 90 min, although the oxidation was incomplete.

**Scheme 1:**
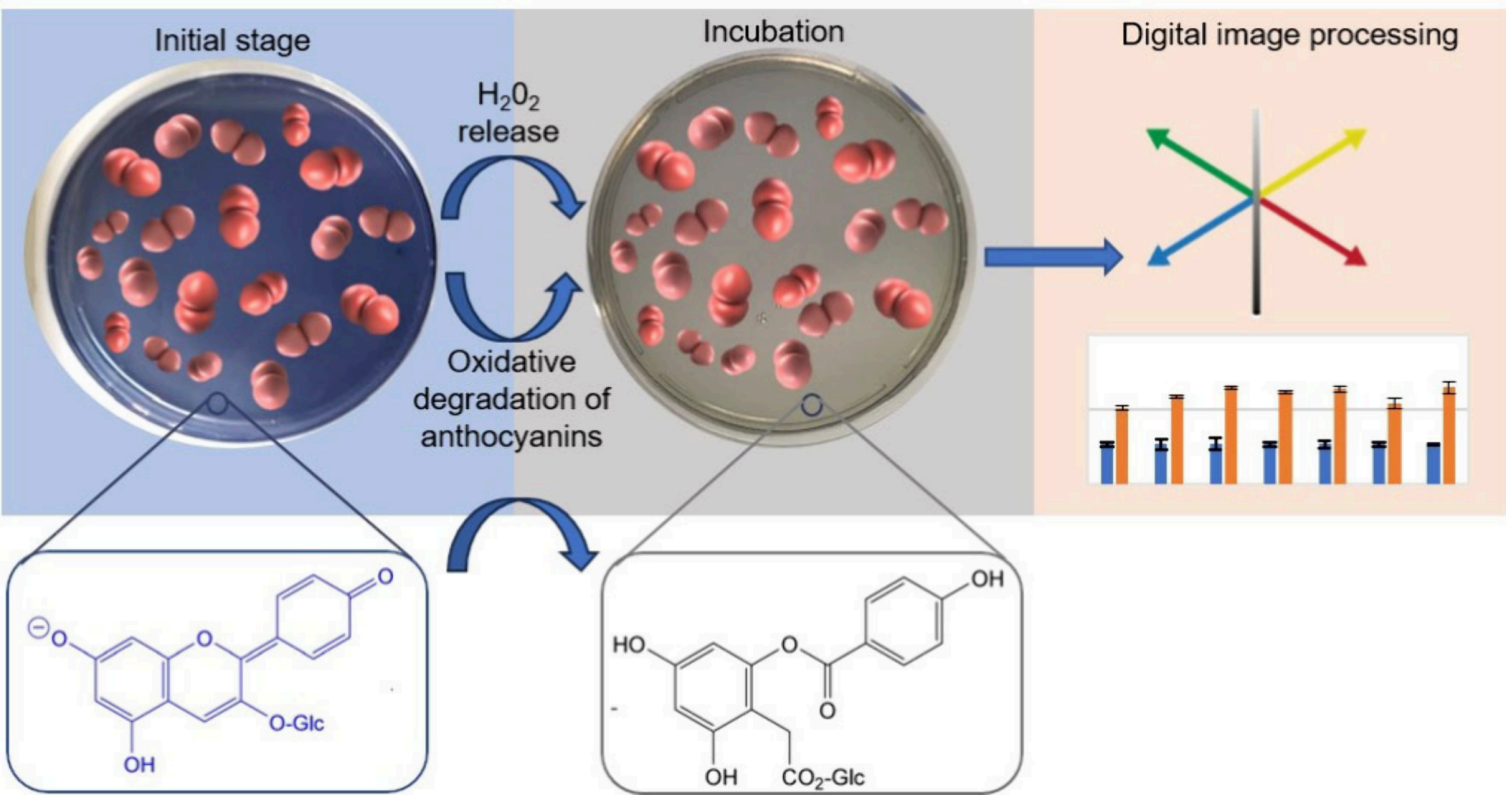
Oxidative Degradation of Anthocyanins in Chromogenic Anthocyanin Agar and the Mechanism of Color Change with H_2_O_2_ Released by *S. pneumoniae*

It should be noted that *S. pneumoniae* grows in the presence of an Optochin disc and produces large quantities of H_2_O_2_. After *S. pneumoniae* was grown under the appropriate conditions, a chromogenic anthocyanin agar was prepared. *S. pneumoniae* was inoculated onto an agar plate and incubated for 24 h (see Figure 1A). Excessively released H_2_O_2_ causes oxidative degradation of anthocyanin on agar plates. The change in color from blue to gray shows that there are bacterial growth and a very high level of H_2_O_2_ is released. Figure 1B and 1C shows the colorimetric results of the chromogenic anthocyanin agar with RGB and ΔE analyses. *S. pneumoniae* was inoculated into two separate areas (area with Optochin disk and without Optochin) of the agar plate, then both areas on the agar were completely turned to gray. The R/B analysis is determined by calculating the ratio of the red to blue values to demonstrate the transformation from blue to gray. As the blue color value decreases during incubation, the R/B ratio increases. Figure 1B shows that this ratio increased because of incubation. The clear distinction between the ΔE values demonstrates that the color change before and after incubation can be more easily identified. Figure 1C presents the ΔE values of the *S. pneumoniae* in the gray agar plate compared to the values before incubation.

**Figure 1:**
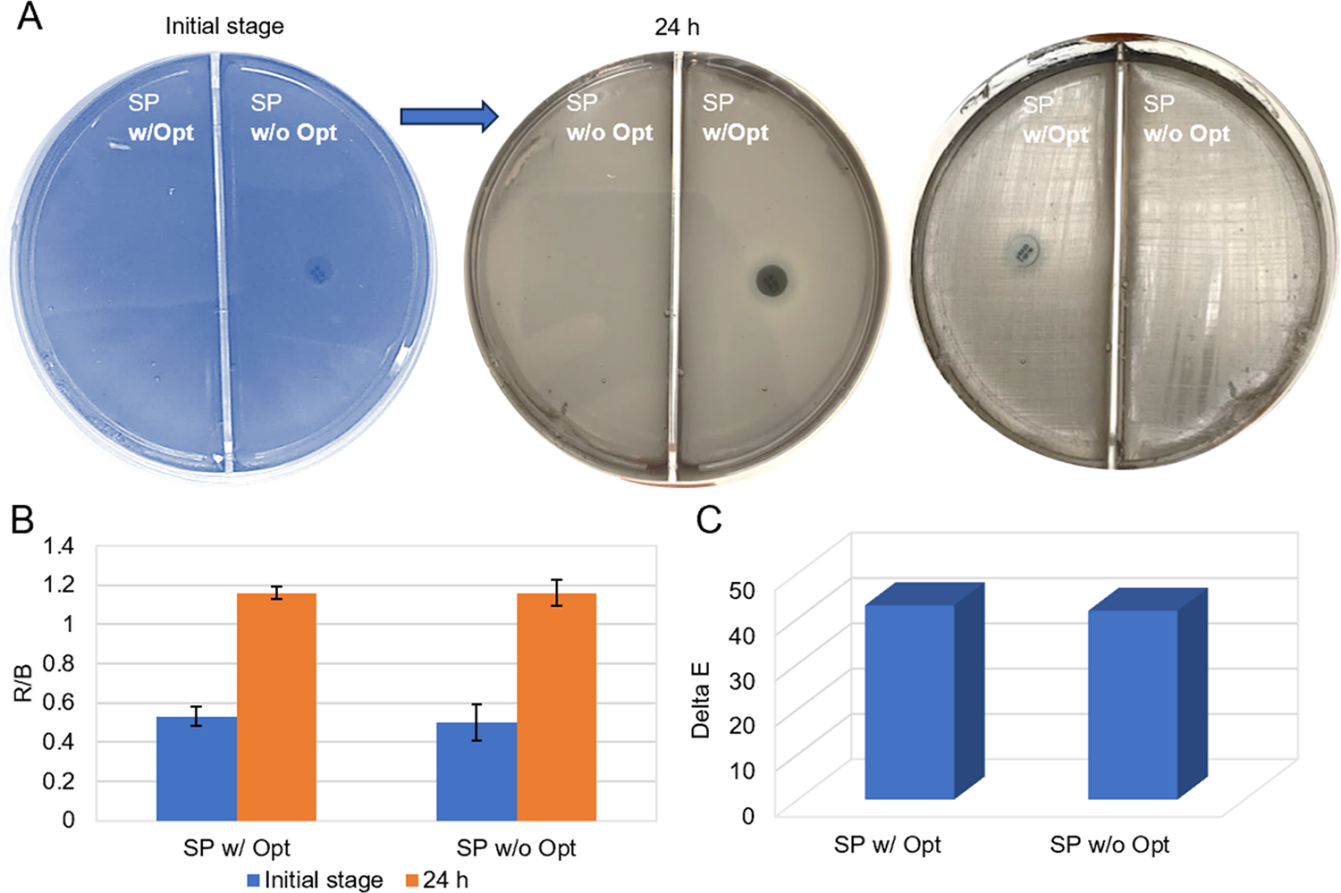
(A) Colorimetric results of the chromogenic anthocyanin agar at the initial stage and after 24 h of incubation were used to detect *S. pneumoniae*, (B) R/B analysis and (C) ΔE values that show the quantitative results of the initial stage and after incubation. The error bars represent standard deviation (SD) generated from three measurements (*n* = 3). (Raw data provided in Table S1.)

To examine the color change of the chromogenic anthocyanin agar depending on time, *S. pneumoniae* (1000 CFU/mL) was inoculated and the gray color of the agar was photographed at specific time intervals. As shown in Figure 2A, no gray color was recorded during the initial 6 h following inoculation. Detailed images for the lag phase (0–6 h) have been added to Supporting Information Figure S2. Mild gray spots were observed on chromogenic anthocyanin agar between 7 and 11 h after inoculation, while a partial gray color was generally observed on the surface of chromogenic anthocyanin agar between 12 and 14 h after inoculation. The high-frequency monitoring between 7 and 14 h demonstrates the rapid colorimetric signal development. The subsequent interval (14 to 24 h) represents the saturation phase, where the colorimetric change reaches its maximum intensity, resulting in the distinct and stable gray color observed across the entire surface of chromogenic anthocyanin agar at the 24 h end point. After 24 h of incubation (the intended readout time), the colorimetric response reached a fully developed gray appearance across the inoculated surface of the chromogenic anthocyanin agar. Importantly, no further noticeable change in the gray appearance was observed upon extended incubation to 30 h under our experimental conditions (Figure S3). The time-dependent color change is supported by R/B analysis in Figure 2B and ΔE analysis in Figure 2C. The color change, which began at the 7th h, reached its maximum level at the 24th h.

**Figure 2:**
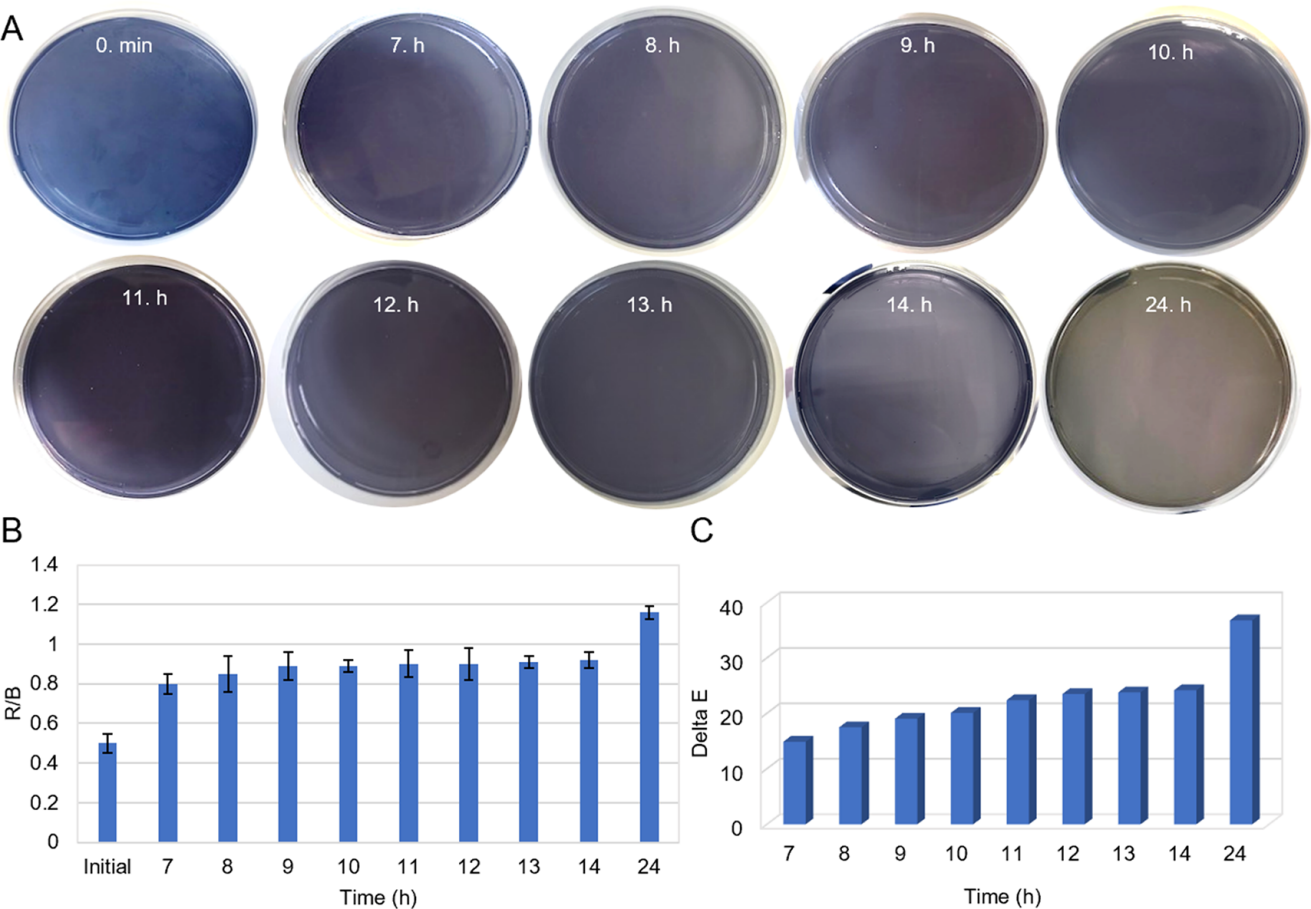
(A) Colorimetric results of time-dependent chromogenic anthocyanin agar after inoculation with 1000 CFU/mL *S. pneumoniae*, (B) R/B analysis and (C) ΔE values. (Raw data provided in Table S2.)

To observe color changes in dependence on bacterial concentration, *S. pneumoniae* strains were inoculated onto chromogenic anthocyanin agar at concentrations of 1, 10, 100, and 1000 CFU/mL and it was kept in the incubator for 24 h. The agar remained stable in the absence of bacterial inoculation, exhibiting no chromatic change from its original blue color (Figure 3A). Meanwhile, the agar containing various concentrations of *S. pneumoniae* (1, 10, 100, and 1000 CFU/mL) turned gray, as illustrated in Figure 3B–E. The color change of the chromogenic anthocyanin agar depending on the *S. pneumoniae* concentration was quantitatively supported by ΔE and RGB analysis. As shown in Figure 3F, the R/B ratio increased in the *S. pneumoniae* inoculated at various concentrations by comparison with the control group. Additionally, 1000 and 1 CFU/mL produced very similar R/B values, indicating that distinct colorimetric results were produced at low concentrations. Figure 3G shows that high ΔE values were recorded for all bacterial concentrations compared to the control agar plate. A systematic approach was used to analyze the detection of *S. pneumoniae*, which was evaluated based on incubation time and colony count. *S. pneumoniae* grew significantly on chromogenic anthocyanin agar in all colonies, including at a concentration of 1 CFU/mL, after a 24 h incubation period. Gray spots appeared after 7 h of incubation, following the inoculation of 1000 CFU/mL of *S. pneumoniae*.

**Figure 3:**
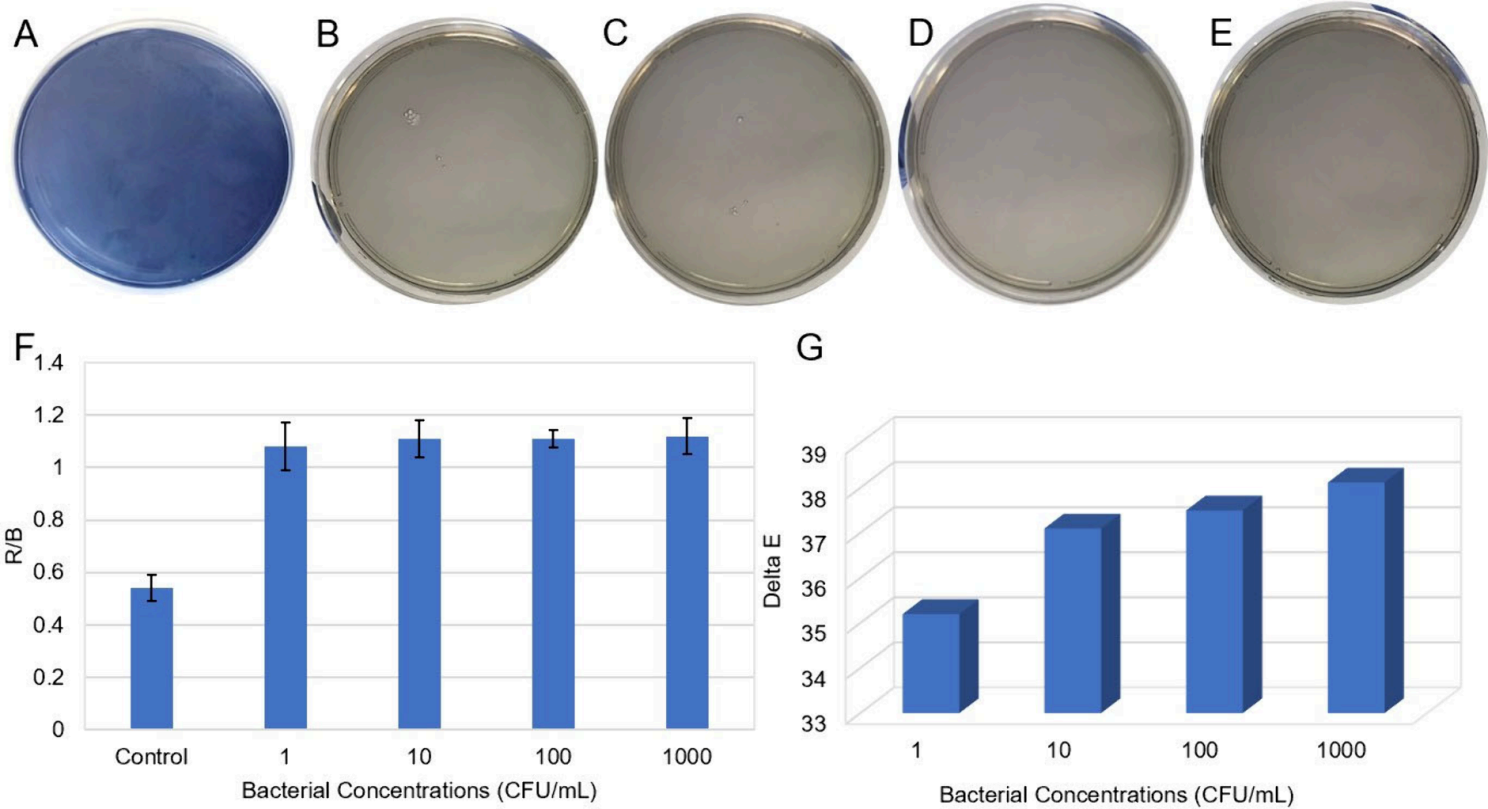
(A) Colorimetric results for bare chromogenic anthocyanin agar and for *S. pneumoniae* inoculated at concentrations of (B) 1, (C) 10, (D) 100 and (E) 1000 CFU/mL on the agar plates, (F) R/B analysis and (G) ΔE values. (Raw data provided in Table S3.)

To analyze the selectivity of chromogenic anthocyanin agar with other bacterial species, the agar plate was divided into seven sections. Each section was inoculated with *S. pneumonia* (SP), *Staphylococcus aureus* (SA), *Enterococcus faecalis* (EFs), *Enterococcus faecium* (EFm), *E. coli* (EC), *Streptococcus agalactiae* (SAg) and *Streptococcus pyogenes* (SPy) strains at 0.5 McFarland (Figure 4A). The *Staphylococcus aureus*, *Enterococcus faecalis*, *Enterococcus faecium*, *E. coli*, *Streptococcus agalactiae* and *Streptococcus pyogenes* strains continued to grow. The purple color of the chromogenic anthocyanin agar turned pink due to the acidic volatile compounds they released into the medium. In contrast, the area inoculated with *S. pneumoniae* turned from blue to gray. Simultaneously, color changes caused by multiple species in the chromogenic anthocyanin agar were observed. The release of H_2_O_2_, a *S. pneumoniae* virulence factor, was proven a strong distinguishing feature in the presence of other species. Comparing the R/B values after incubation with the initial R/B ratios showed that *S. pneumoniae* exhibited the lowest R/B value (Figure 4B). Much higher R/B values were observed in other bacterial species, as this value increases significantly during the transformation from blue to pink. However, during the transition from blue to gray, the increase was limited and less distinct than the increase to pink. In the ΔE analysis, *S. pneumoniae* exhibited a notable increase compared to other bacteria due to the color difference that occurred during the transition from blue to gray (Figure 4C). The creation of a distinctive color difference means that *S. pneumoniae* can be clearly distinguished from other species in chromogenic anthocyanin agar.

**Figure 4:**
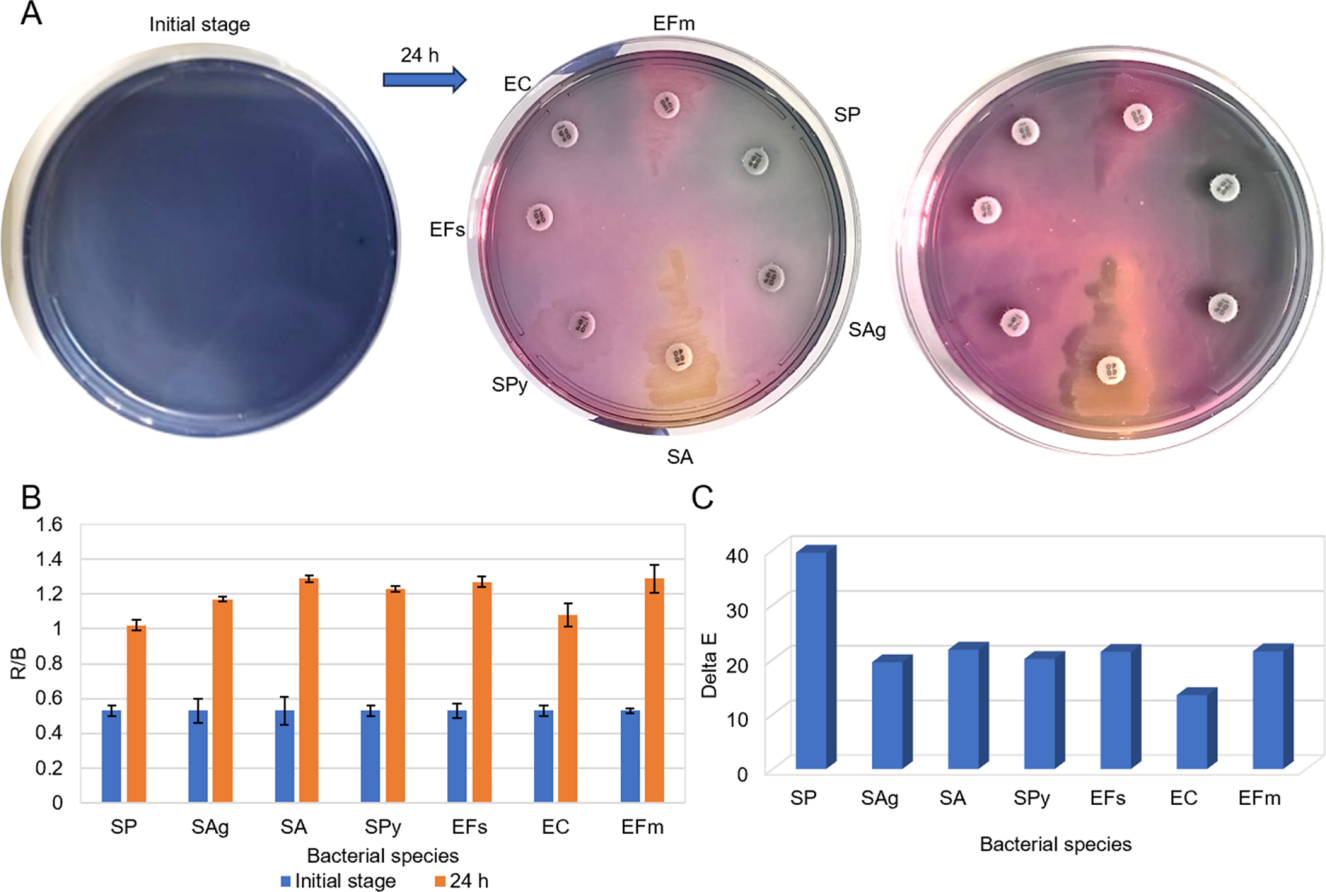
(A) Colorimetric results of a series of different bacterial species inoculated on the agar plates, (B) R/B analysis and (C) ΔE values. (Raw data provided in Table S4).

To demonstrate the clinical utility of the developed method, a comparative analysis with existing diagnostic modalities is presented in Table 1. As highlighted in the table, while commercial chromogenic agars often exhibit inadequate recovery for *S. pneumoniae* ^19^ the anthocyanin chromogenic agar offers a superior alternative by providing visible detection within just 7 h at a significantly lower cost. This confirms that the proposed medium fills a critical gap for rapid screening in resource-constrained settings where expensive molecular infrastructure is unavailable.

**Table 1:** Comprehensive Comparison of the Proposed Anthocyanin Chromogenic Agar with Conventional, Molecular, and Commercial Diagnostic Methods for *S. pneumoniae* and Other Pathogens

Diagnostic Method	Principle/Mechanism	Detection Time	Cost & Complexity	Performance/Limitations	References
Standard Culture (Blood Agar)	Alpha-hemolysis & Secondary Tests (Optochin, Bile Solubility)	24–48 h	Low cost, but labor-intensive	Requires subjective interpretation and additional confirmatory tests. Standard practice but long process.	^18^
Molecular Methods (PCR, MALDI-TOF)	Genomic or Proteomic Identification	2–4 h	Very high cost; requires specialized infrastructure	High sensitivity/specificity. Not feasible for routine screening in resource-limited settings.	^23^
Immunochromatographic Tests (ICT)	Urinary Antigen Detection (Cell wall polysaccharides)	15 min	High cost per test	Rapid but nonculture based. Moderate sensitivity.	^44^
Commercial Chromogenic Agars (General Use)	Synthetic Enzyme Substrates	18–24 h	Moderate/High Cost	Successful for other pathogens: *E. coli*, *K. pneumoniae*, and *E. faecium,* etc.	^28^
Specific Commercial Agars (Chromatic MH agar) for 24 bacterial and 3 yeast species	Synthetic Substrates	18–24 h	High Cost	Inadequate Recovery: Recent studies report failure to recover *S. pneumoniae* or need for formulation optimization.	^29^
Anthocyanin Chromogenic Agar (Present Work)	Oxidative Degradation (H_2_O_2_ mediated)	7–24 h (Visible signal at 7 h)	Low cost; uses natural pigment	Rapid and High Selectivity. Specific ″gray/colorless″ zones distinguish *S. pneumoniae* without secondary tests. Addresses the gap mentioned by Robberts et al.	Present Study

### Challenges and Recommendations for Future Works

Although the anthocyanin-based chromogenic agar presents a rapid and cost-effective alternative for *S. pneumoniae* detection, several challenges must be addressed to facilitate its transition from a proof-of-concept to a commercial diagnostic product. The primary challenge lies in the inherent instability of natural anthocyanins. Unlike synthetic chromogens, anthocyanins are susceptible to degradation due to pH fluctuations, temperature, and light exposure, which can affect the shelf life and consistency of the agar plates. The batch-to-batch variation in crude plant extracts further complicates industrial scale-up and standardization. To overcome this, future studies should focus on the purification of specific high-stability anthocyanins like acylated anthocyanins or the application of microencapsulation technologies to protect the pigment until bacterial interaction occurs. This would ensure a commercially viable shelf life comparable to synthetic media.

The present study validated the method using standard ATCC reference strains. However, *S. pneumoniae* exhibits significant genetic diversity across its more than one hundred serotypes, which may influence H_2_O_2_ production rates. There is a theoretical risk that certain nonencapsulated strains or specific serotypes might produce insufficient H_2_O_2_ to trigger the color change within 7 h. Therefore, large-scale multicenter validation studies using a diverse library of fresh clinical isolates are imperative to confirm the diagnostic sensitivity across different genetic backgrounds.

## 3 Conclusion

Chromogenic anthocyanin agar has been successfully used for both the culture and diagnosis processes of *S. pneumoniae*. We have demonstrated the H_2_O_2_-mediated oxidative degradation of anthocyanin in chromogenic anthocyanin agar through experimental and systematic analysis. The results were demonstrated using both colorimetric and quantitative analysis rely on the RGB color model. In contrast to conventional chromogenic diagnostic agars, chromogenic anthocyanin agar can reduce both incubation time and cost because it performs culture and diagnosis simultaneously. In conclusion, we claim that chromogenic anthocyanin agar has shown great promise in the detection of *S. pneumoniae* and could potentially be used in clinical practices.

## 4 Experimental Section

### Materials and Instruments

Tryptic soy agar, skimmed milk medium, beef extract, and agar were purchased from Thermo Scientific Oxoid (UK). Sodium chloride (NaCl) and hydrogen peroxide were obtained from Isolab (Türkiye), while peptone was purchased from Mast Diagnostic (UK).

### Microorganisms

*Streptococcus pneumoniae* ATCC 49619, *Staphylococcus aureus* ATCC 29213, *Escherichia coli* ATCC 25922, *Enterococcus faecalis* ATCC 29212, *Enterococcus faecium* ATCC 8459, *Streptococcus agalactiae* ATCC 12401, *Streptococcus pyogenes* ATCC 19615 were obtained from Bandirma Onyedi Eylul University, Vocational School of Health Services. Stock cultures were maintained frozen at −20 °C using skimmed milk as a preservative medium and they were recultured in triptic soy agar before the experiments. The optic densities of the bacterial suspensions in saline were determined using a spectrophotometer.

#### Safety Statement

Researchers completed all microbiological experiments involving *Streptococcus pneumoniae* in a Biosafety Level 2 (BSL-2) laboratory. Hydrogen peroxide solutions were used with appropriate personal protective equipment to prevent skin and eye contact.

### Preparation of Red Cabbage Extract

Red cabbage (*Brassica oleracea* L., family Brassicaceae) is a natural source of anthocyanins. These anthocyanins are glycosides of 2-phenylbenzopyrylium or flavilium salts. The main anthocyanins found in Brassica plants are derivatives of cyanidin 3-diglucoside-5-glucoside that are linked with various aromatic and aliphatic acids, as well as with glucosides and xylose. To extract anthocyanins from red cabbage, first the purple leaves are separated from the cabbage, then cleaned and dried. The next step is the cutting of the dried leaves into small pieces. One hundred grams of the cut leaves are boiled in 100 mL of distilled water for 30 min. After this 30 min period, we filter the extracted purple solution through Whatman No. 1 filter paper. The liquid that has been filtered is kept in amber-colored glass bottles at a temperature of 4 °C so that it can be used in experiments.^45,46^

### Preparation of Chromogenic Anthocyanin Agars

To prepare the 2X growth medium (GM), 20 g/L peptone, 2 g/L beef extract, 30 g/L agar and 150 g/L salt were sterilized in an autoclave at 121 °C for 15 min.^32^ A 10% solution of red cabbage extract was prepared, and the pH was adjusted to 8.0 using a 1 M NaOH solution. The blue anthocyanin solutions were sterilized using the filtration method. The GM and anthocyanin solutions were mixed at a 1:1 ratio. This mixture was divided between plates. Bacterial suspensions were prepared in saline and adjusted using a McFarland densitometer. The bacterial strains were inoculated onto the plates using a sterilized loop. The chromogenic anthocyanin agar plate was incubated at 37 °C and the color change over time was recorded.

### Digital Image Processing

Digital image processing was used to accurately distinguish color changes. The observation process requires additional steps if it is to be successful. At the end of the incubation period, photographs of each diagnostic agar were taken and analyzed using ImageJ software (National Institutes of Health). ImageJ software was used to analyze the red, green and blue channels of the colorimetric changes in the agar plate. The red/blue ratio was calculated to demonstrate the formation of a gray color by pneumococci compared to other bacterial strains. ΔE analysis was also performed to measure the difference between the two colors. The CIE 1976 Lab formula was used to measure color differences.^47,48^

$$\Delta E={\left[{\left(\Delta L\right)}^{2}+{\left(\Delta a\right)}^{2}+{\left(\Delta b\right)}^{2}\right]}^{1/2}$$ 1

The ΔE values were calculated based on the three fundamental differences defined in Formula [Disp-formula eq1]: ΔL, Δa, and Δb. The dimensions of the CIE Lab color space are defined by these three elements. The difference between red and green is represented by Δa, the difference between yellow and blue by Δb, and the difference between black and white by ΔL. Basically, these differences are low when it is similar images and high when it is different images.^49,50^ This analysis allows for more accurate color difference analysis in the diagnostic agars than is possible with the naked eye.

## Supplementary Material



## Notes

Photographs of anthocyanin solutions exposed to varying H_2_O_2_ concentrations and colorimetric results of the anthocyanin chromogenic agars; raw colorimetric data (PDF)
