# Supplementary material for: Anthocyanin-Incorporated Chromogenic Agar for Rapid, Selective Detection of Streptococcus pneumoniae via Hydrogen Peroxide-Mediated Oxidation
Source: ACS Omega. 2026 Mar 2;11(10):16806–15. doi: 10.1021/acsomega.5c13596 (PMC13000601; doi:10.1021/acsomega.5c13596)
Supplement: Supplementary file 1 [file ao5c13596_si_001.pdf]

Supporting Information

Anthocyanin-Incorporated Chromogenic Agar for Rapid, Selective Detection of *Streptococcus pneumoniae* via Hydrogen Peroxide-Mediated Oxidation

Cagla Celik Yoldas[a]*, *Nimet Temur*[b], *Nilay Ildiz*[c], *Pinar Sagiroglu*[d], *Mustafa Altay Atalay*[d], *Ismail Ocsoy*[b]

[a]Department of Analytical Chemistry, Faculty of Pharmacy, Harran University, 63200, Sanliurfa, Türkiye

[b]Department of Analytical Chemistry, Faculty of Pharmacy, Erciyes University, 38039, Kayseri, Türkiye

[c]Medical Imaging Department, Vocational School of Health Services, Bandirma Onyedi Eylul University, 10200, Balikesir, Türkiye

[d]Department of Medical Microbiology, Faculty of Medicine, Erciyes University, 38039, Kayseri, Türkiye

*E-mail: caglacelik@harran.edu.tr

1. Results and Discussion

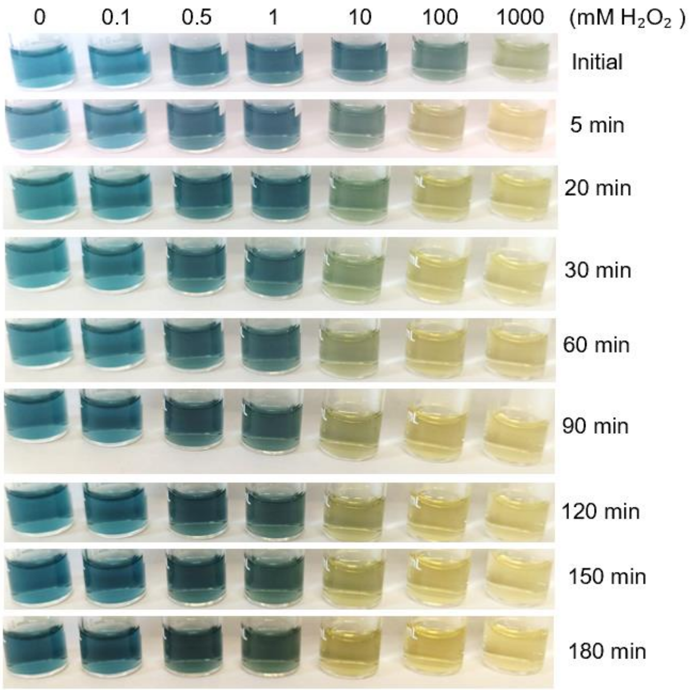

Figure S1. Concentration-dependent colorimetric response of anthocyanin to H_2O_2.

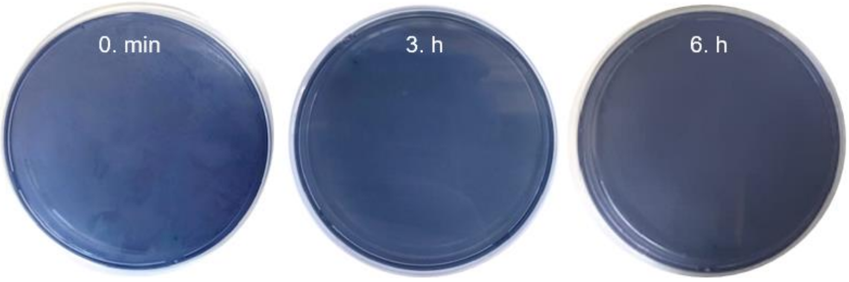

Figure S2. Colorimetric results of the anthocyanin chromogenic agar after inoculation with 1000 CFU/mL *S. pneumonia* during the initial incubation period.

Images captured at 0, 3, and 6 hours (h) following inoculation with *S. pneumoniae* show no discernible color change, with the agar retaining its original color. This confirms that oxidative degradation of anthocyanins does not occur during the bacterial lag phase, as H_2O_2 production is insufficient to change the colorless form of anthocyanins.

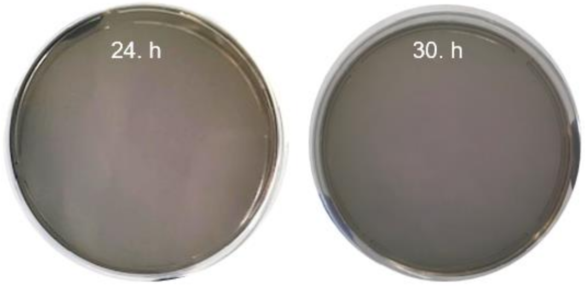

Figure S3. The colorimetric results of 24. h and 30. h chromogenic anthocyanin agar after inoculation with 1000 CFU/mL *S. pneumonia*.

Images at 0 min, 24 h, and 30 h after inoculation with *S. pneumoniae* indicate that the gray color is fully developed at 24 h and remains visually stable up to 30 h under our experimental conditions.

Table S1. Raw data corresponding to the colorimetric measurements shown in Figure 1.

**R/B**	Initial stage	24 h
SP w/ Opt	0.53	1.16
SP w/o Opt	0.5	1.16

**Delta E**	24 h
SP w/ Opt	42.9
SP w/o Opt	41.7

Table S2. Raw data corresponding to the colorimetric measurements shown in Figure 2.

**R/B**	Initial stage	7. h	8. h	9. h	10. h	11. h	12. h	13. h	14. h	24. h
SP	0.5	0.8	0.85	0.89	0.89	0.9	0.9	0.91	0.92	1.16

**Delta E**	7. h	8. h	9. h	10. h	11. h	12. h	13. h	14. h	24. h
SP	14.9	17.5	19.06	20.1	22.4	23.51	23.81	24.2	36.8

Table S3. Raw data corresponding to the colorimetric measurements shown in Figure 3.

**R/B**	Control	1 CFU/mL	10 CFU/mL	100 CFU/mL	1000 CFU/mL
SP	0.54	1.08	1.11	1.11	1.12

**Delta E**	1 CFU/mL	10 CFU/mL	100 CFU/mL	1000 CFU/mL
SP	35.2	37.1	37.5	38.12

Table S4. Raw data corresponding to the colorimetric measurements shown in Figure 4.

**R/B**	Initial stage	24 h
SP	0.53	1.02
SAg	0.53	1.17
SA	0.53	1.29
SPy	0.53	1.23
EFs	0.53	1.27
EC	0.53	1.08
EFm	0.53	1.29

**Delta E**	24 h
SP	39.5
SAg	19.5
SA	21.8
SPy	20.1
EFs	21.4
EC	13.5
EFm	21.5
